# An Unusual Delayed Recurrence of Temporal Ameloblastoma: A Case Report

**DOI:** 10.7759/cureus.113279

**Published:** 2026-07-24

**Authors:** Abira Chattopadhyay, Nayana De, Aritra Chatterjee, Md Arif Hossain, Basabdatta Ghosh

**Affiliations:** 1 Oral and Maxillofacial Surgery, Dr. R. Ahmed Dental College and Hospital, Kolkata, IND

**Keywords:** ameloblastoma, coronoid process, odontogenic tumour, preauricular mass, recurrence

## Abstract

Ameloblastoma is a benign but locally aggressive odontogenic tumor with a recognized tendency for recurrence. Recurrence after more than a decade is exceedingly rare. We report a case of a 46-year-old woman who presented with a firm, non-tender, gradually progressive swelling in the left preauricular region of one-year duration. Fine-needle aspiration cytology (FNAC) revealed a lymphocytic infiltrate. Computed tomography demonstrated a soft-tissue lesion adjacent to a bony remnant resembling a coronoid process. Histopathology confirmed follicular ameloblastoma. Surgical excision of the lesion with the coronoid process remnant was performed under general anesthesia. The postoperative period was uneventful, and no early recurrence was noted.

## Introduction

Ameloblastoma is a benign odontogenic epithelial neoplasm characterized by slow growth, local invasiveness, and a marked propensity for recurrence despite its benign histologic appearance. It represents one of the most common clinically significant odontogenic tumors and most frequently involves the mandible [1,2]. Histopathologically, the follicular subtype is among the most frequently encountered variants and has been associated with a greater tendency to recur than certain other patterns, with a recurrence rate of around 29.5% [3,4].

Recurrence of ameloblastoma most commonly occurs within the first five years following treatment; however, delayed recurrence several years or even decades after initial management has been documented [5,6]. Proposed mechanisms include residual microscopic disease, persistence of tumor islands within the adjacent soft tissue or bone, and the lesion's inherently infiltrative growth pattern [7]. Such late presentations highlight the need for prolonged surveillance even in patients who have remained asymptomatic for extended periods.

Diagnosis of recurrent ameloblastoma may be challenging when lesions arise in atypical locations or present predominantly as soft-tissue masses. Cytological investigations may be inconclusive when sampling yields inflammatory, cystic, or reactive components rather than the representative odontogenic epithelium [8]. We report a rare case of follicular ameloblastoma recurring 16 years after hemimandibulectomy, presenting as a preauricular mass associated with a remnant of the coronoid process.

## Case presentation

**Figure 1:**
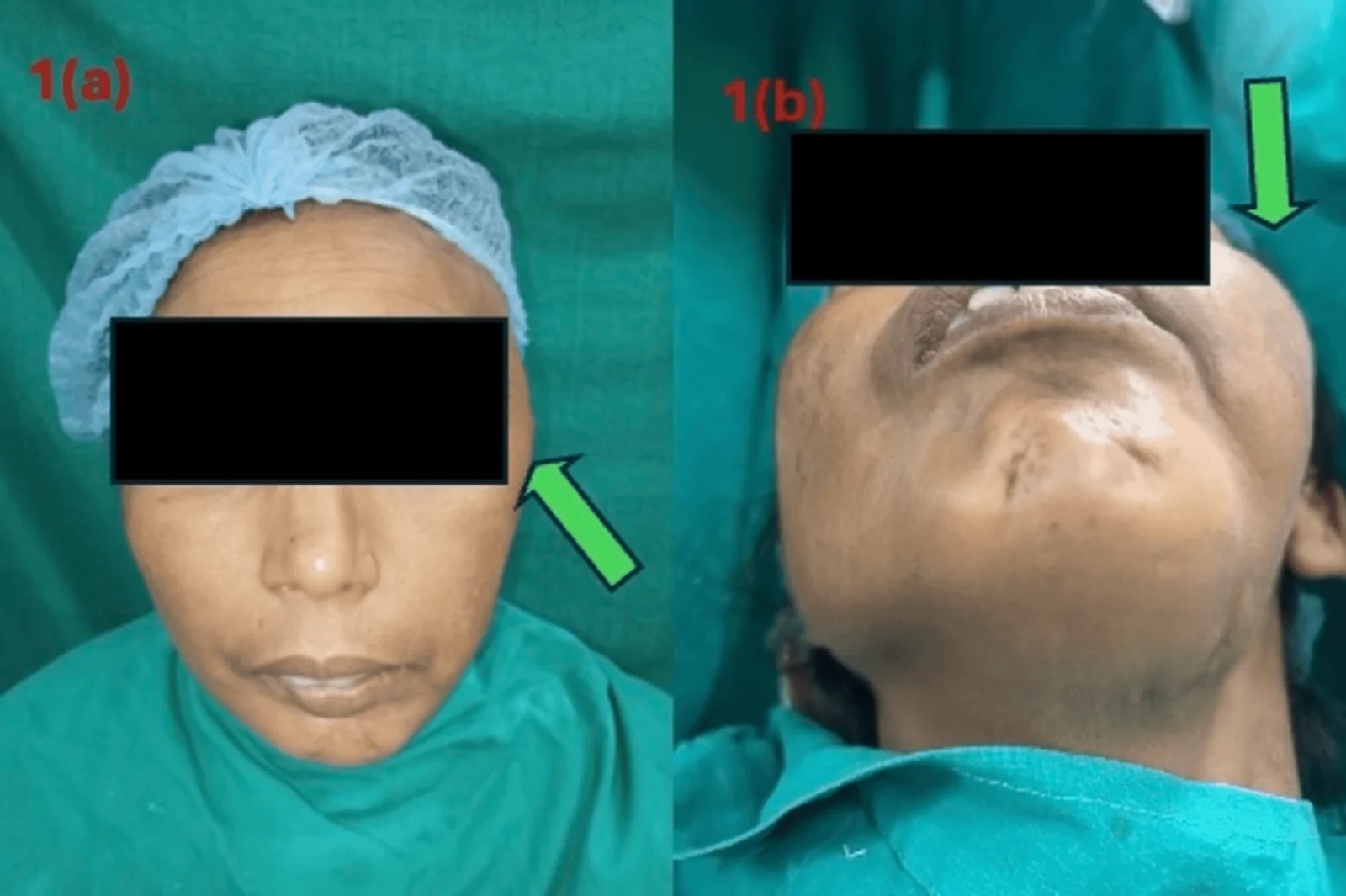
Pre-operative clinical photograph of the patient presenting with temporal swelling The patient presented with a gradually enlarging temporal swelling 16 years after surgical treatment (hemimandibulectomy) of mandibular ameloblastoma. (a) Frontal view demonstrating fullness in the left temporal region (green arrow). (b) Inferior view illustrating the contour deformity produced by the lesion (green arrow).

A 46-year-old woman presented with a gradually enlarging swelling in the left temporal region of one year's duration (Figure 1). The swelling was non-tender, firm, and non-compressible. The patient had undergone a left hemimandibulectomy for ameloblastoma 16 years earlier and had remained asymptomatic during the intervening period.

Clinical examination demonstrated a well-defined preauricular swelling with normal overlying skin. No facial nerve dysfunction, trismus, or significant limitation of mandibular movement was noted. Intraoral examination revealed satisfactory mouth opening and a stable occlusion.

**Figure 2:**
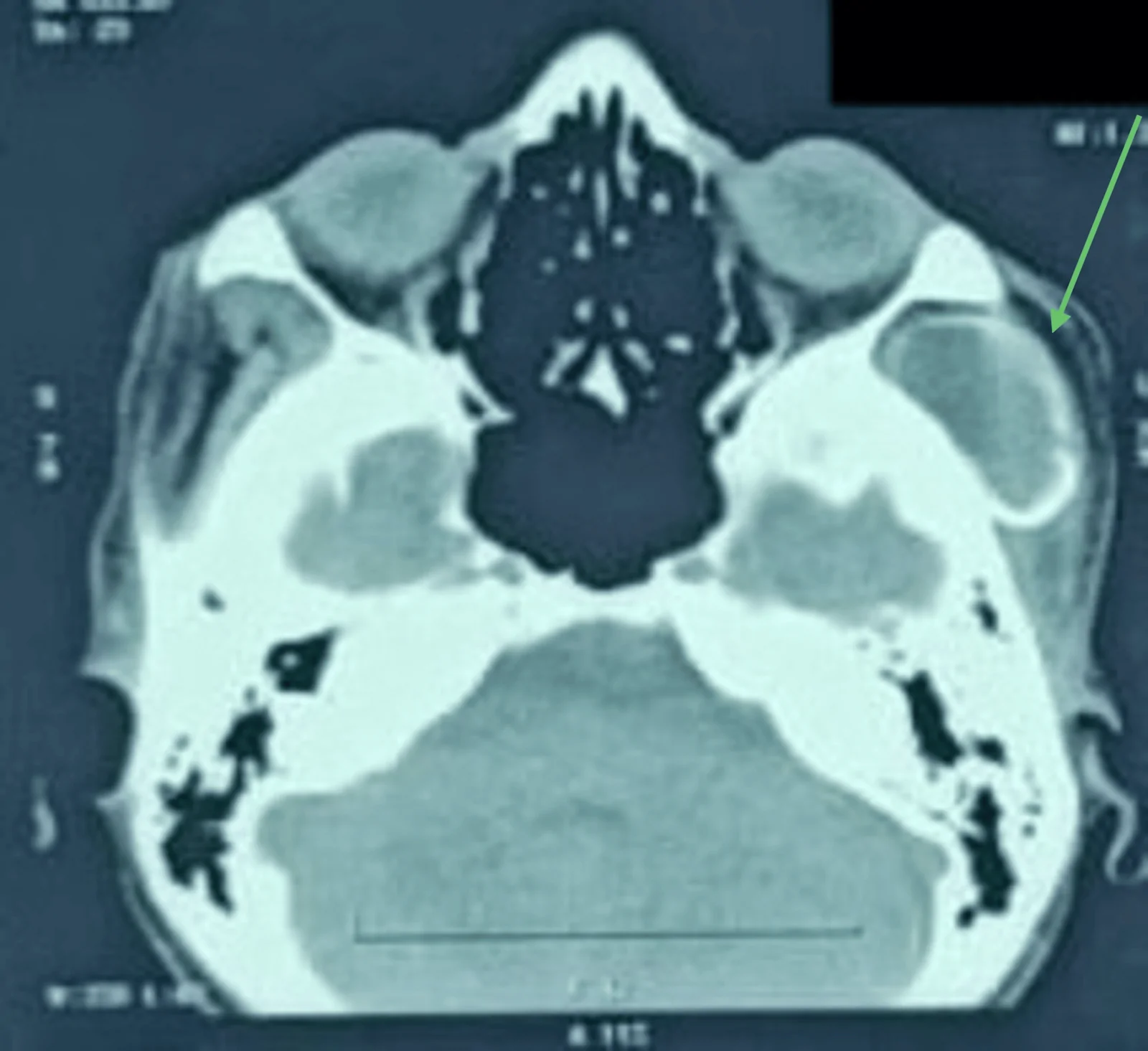
Computed tomography of the face showing a space-occupying lesion in the left temporal region Axial computed tomography images showing a well-defined space-occupying lesion in the temporal region (green arrow), closely associated with what appears to be a bony structure resembling the residual coronoid process. Radiographic findings raised a suspicion of recurrent pathology because of a history of hemimandibulectomy for ameloblastoma.

Fine-needle aspiration cytology (FNAC) was inconclusive, showing predominantly lymphocytic inflammatory cells. Computed tomography revealed a soft-tissue lesion intimately associated with a bony structure resembling a remnant of the coronoid process (Figure 2).

**Figure 3:**
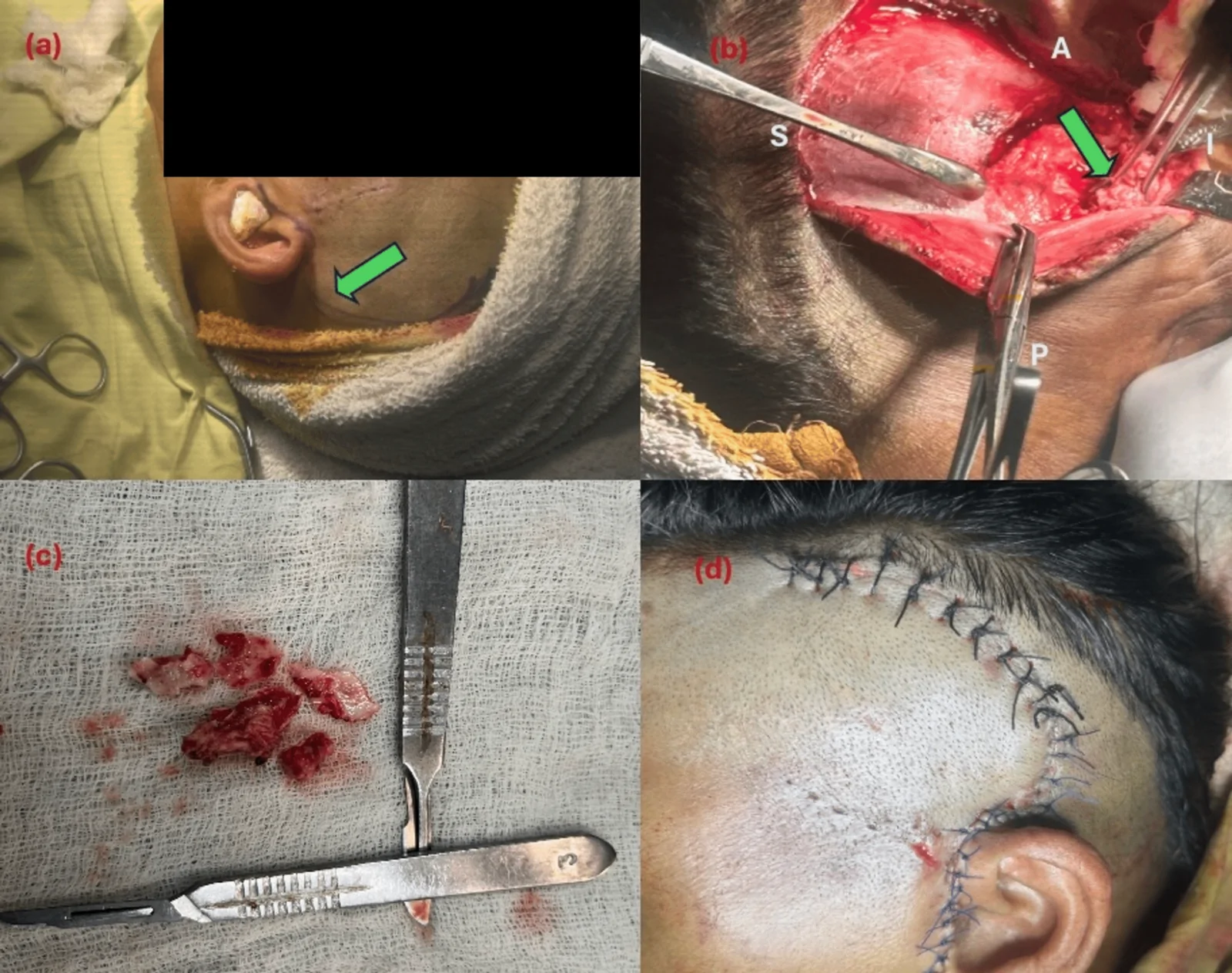
Intraoperative findings and surgical management of recurrent ameloblastoma (a) Preoperative marking of the lesion in the left preauricular region (green arrow). (b) Intraoperative exposure of the lesion through a preauricular approach. The recurrent tumor mass is indicated by the green arrow (A, anterior; P, posterior; S, superior; I, inferior). (c) Excised specimen following en bloc resection. (d) Immediate postoperative view demonstrating wound closure.

Surgical exploration under general anesthesia revealed a firm mass attached to the remnant of the coronoid process. En bloc excision of the lesion together with the associated bony fragment was performed (Figure 3).

**Figure 4:**
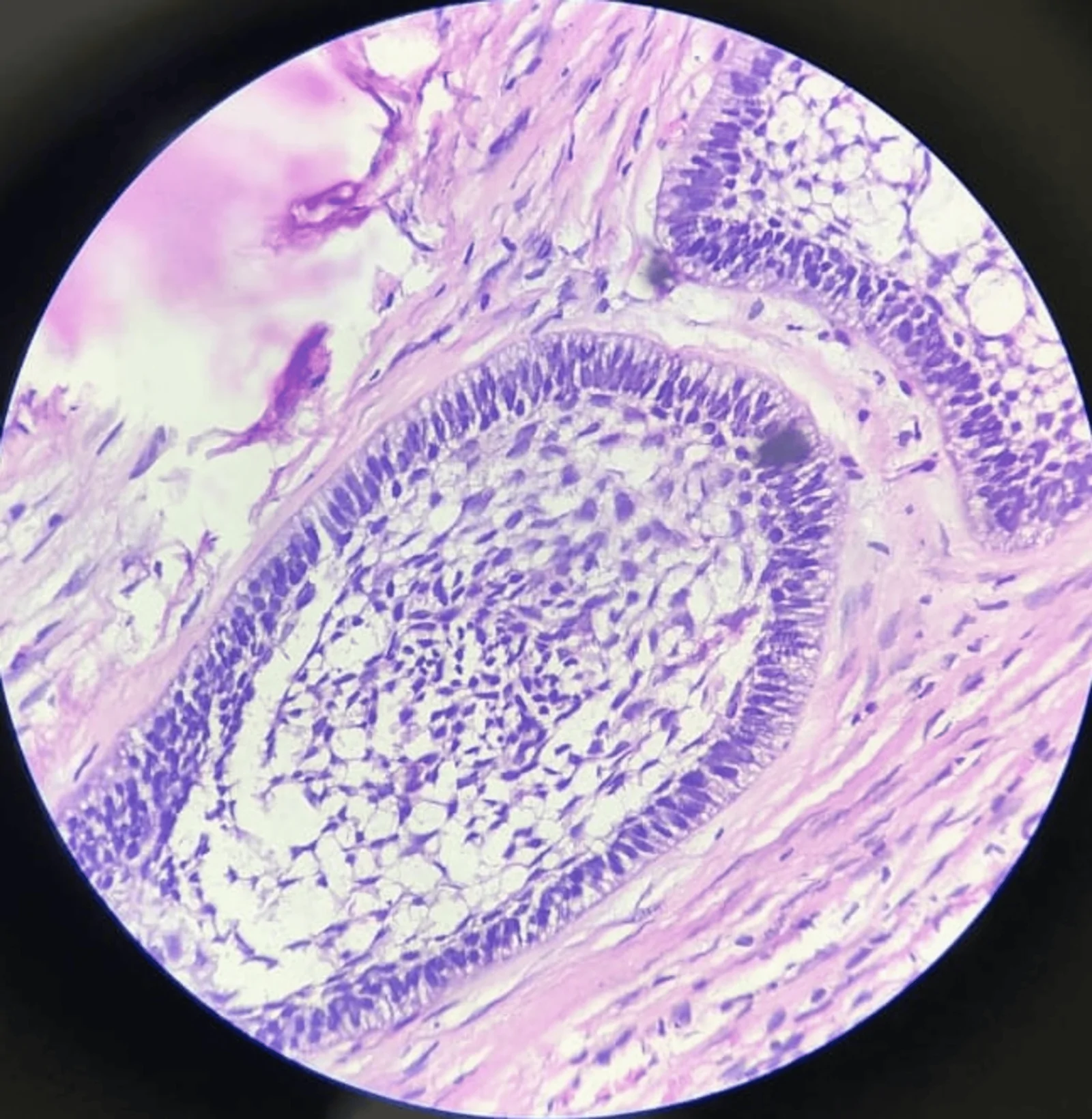
Histopathological features of recurrent follicular ameloblastoma Odontogenic epithelial islands exhibiting peripheral palisading of columnar ameloblast-like cells with reverse nuclear polarity and central stellate reticulum-like cells, consistent with the follicular variant of ameloblastoma (H&E stain, magnification 40X).

Histopathological examination demonstrated nests and islands of odontogenic epithelium with peripheral palisading, reverse nuclear polarity, and central stellate reticulum-like areas, confirming the diagnosis of follicular ameloblastoma (Figure 4).

**Figure 5:**
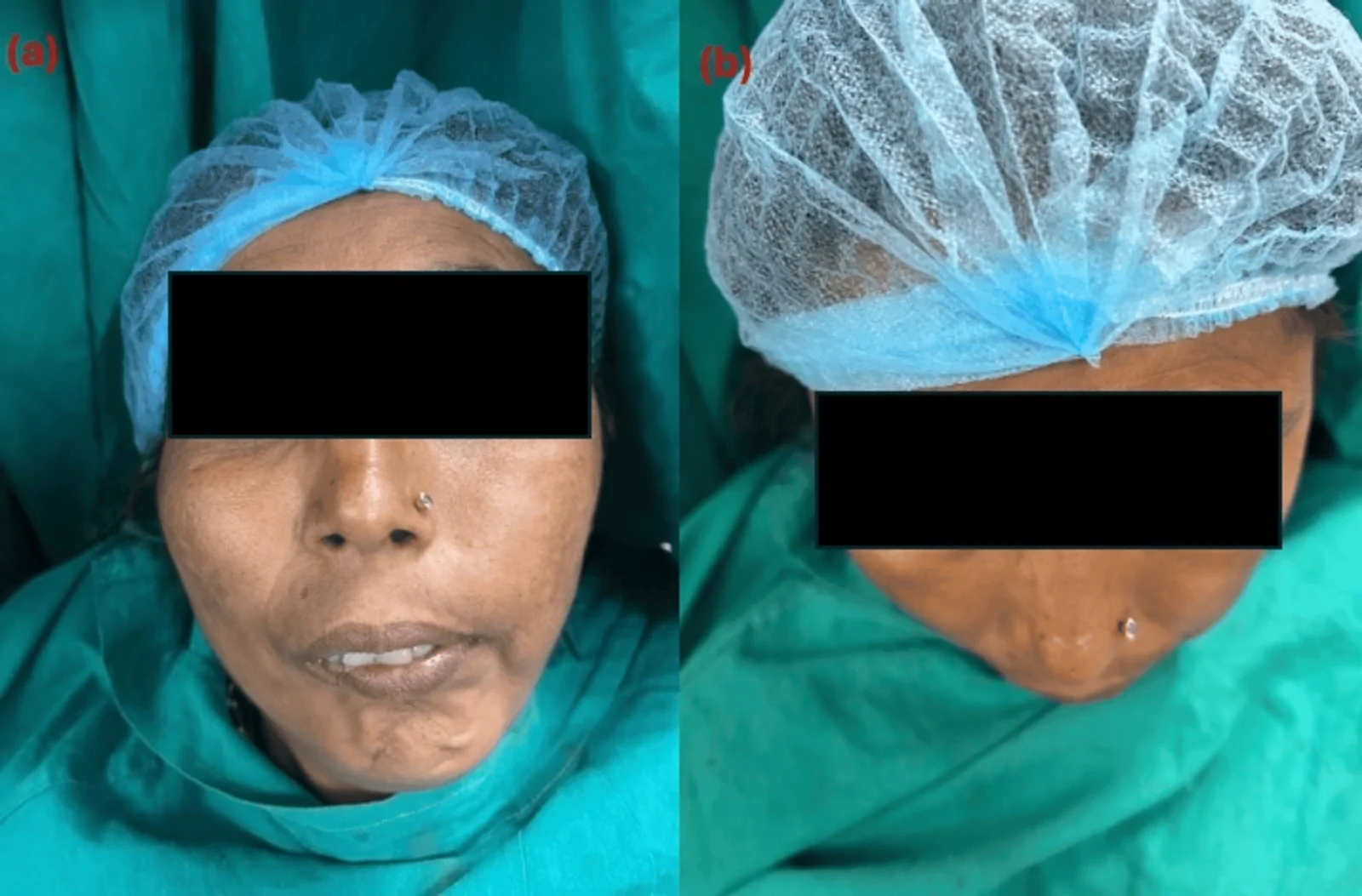
One-year postoperative follow-up showing satisfactory healing without evidence of recurrence (a) Frontal view at the one-year follow-up demonstrating satisfactory facial symmetry and absence of clinically appreciable swelling in the left preauricular region. (b) Superior (bird’s-eye) view at the one-year follow-up showing a stable postoperative contour with no evidence of local recurrence. The postoperative course was uneventful, and the patient remained asymptomatic throughout the follow-up period.

The postoperative course was uneventful. The patient was advised regarding the possibility of further recurrence and the importance of long-term follow-up. At the one-year review, no clinical or radiographic evidence of recurrence was identified (Figure 5).

## Discussion

Although histologically benign, ameloblastoma is known for its locally aggressive behavior and high potential for recurrence [1,2], and the histopathological subtype, extent of disease, and adequacy of the initial surgical treatment influence recurrence rates. Conservative treatment approaches such as enucleation or curettage have consistently demonstrated higher recurrence rates than radical resection with appropriate margins [7,9]. Nakamura et al. have reported a recurrence rate of 33.3% among patients who received conservative management compared to 7.1% recurrence rates in the resection group [10]. In a more recent study, the conservative treatment group demonstrated a recurrence rate of 90.9% [11].

Delayed recurrence beyond 10 years is uncommon but well documented in the literature [4,5]. Such presentations are thought to result from microscopic residual disease that remains clinically silent for prolonged periods before demonstrating renewed growth. The present case is unusual because recurrence became evident 16 years after the initial surgery and presented primarily as a soft-tissue mass in the preauricular region.

The atypical location contributed to the diagnostic challenge. In addition, FNAC failed to establish a definitive diagnosis, yielding only inflammatory cellular elements. Similar limitations of cytological evaluation have been reported in odontogenic tumors, particularly when the sampled area does not contain representative epithelial tumor tissue [8].

The follicular histological pattern identified in the present case has historically been associated with a greater tendency toward recurrence than some other variants [3]. Complete surgical excision remains the cornerstone of treatment, while long-term clinical and radiological surveillance is essential because recurrence may occur many years after apparently successful therapy [4-6,8-12].

This case emphasizes the importance of maintaining a high index of suspicion when evaluating new preauricular or mandibular swellings in patients with a history of ameloblastoma, regardless of the interval since initial treatment.

## Conclusions

Ameloblastoma may recur many years after definitive treatment, underscoring its unpredictable biological behavior. The present case demonstrates a rare 16-year delayed recurrence presenting as a preauricular mass associated with a coronoid process remnant. Clinicians should consider recurrent ameloblastoma in the differential diagnosis of atypical facial swellings in previously treated patients. Complete surgical excision and long-term follow-up remain essential for optimal management.
